# By Residents, for Residents: Evaluating a Community-Led Peer Health Education Program in Australian Social Housing Communities

**DOI:** 10.5334/ijic.9102

**Published:** 2025-11-05

**Authors:** Esther Tordjmann, Fiona Haigh

**Affiliations:** 1 School of Population Health, University of New South Wales, Sydney, Australia; 2 Sydney Local Health District, Australia; 3 International Centre for Future Health Systems, University of New South Wales, Sydney, Australia

**Keywords:** peer education, social housing, community-based health promotion, health equity, co-production, realist evaluation

## Abstract

**Introduction:**

Socially disadvantaged and marginalised groups experience significant barriers to accessing healthcare compounded by the complexity and lack of integration between health services. Social housing residents face persisting health inequities linked to poverty, stigma and chronic conditions. International evidence suggests tailored, community-based initiatives can advance health and equity by building capacity, trust and engagement, notably through peer-based roles. However, there is little detailed literature on the mechanisms that support successful implementation and impacts across both community and health systems.

**Description:**

The paper describes the implementation and evaluation of a community-driven and co-designed peer-to-peer health education program in social housing communities in Australia. We use a mixed-methods, realist-informed methodology to assess the program’s effectiveness and identify success factors at individual, program and organisational levels.

**Discussion:**

An important lesson is that a strength-based and community-led model is effective in areas of entrenched disadvantage and can have greater reach than other health promotion approaches. The program’s flexibility, holistic remit, and focus on sharing power and trust-building were crucial for its success.

**Conclusion:**

Peer and community-based initiatives are becoming an increasingly important component of integrated care programs. Valuing the process as well as the outcomes and providing long-term timeframes and ongoing resources are critical to sustain change.

## Introduction

Socially disadvantaged and marginalised groups experience barriers to accessing healthcare compounded by complexity and lack of integration between health services. Socio-economic deprivation has a strong correlation with poor health outcomes [1] and the scale of a society’s income inequality is also a determinant of population health [2]. Despite broad-based strategies to provide better integrated care and transitions between services such as policies (e.g. NSW Health Strategic Framework for Integrating Care), service models (e.g. Primary Health Networks), physically co-located integrated services [3], and place-based care coordination models [4], inequities persist including in high-income countries with universal public medical cover like Australia [5].

International experience suggests that person-centred and community-based interventions using navigators, community health workers, and other peer-based roles can have an important role in linking marginalised population groups to health and social care services [6,7,8]. While models differ, there is growing evidence that these roles can increase access to health services and cost effectiveness [9,10,11]. Because these roles often navigate across health and social care, they tend to engage beyond the clinical community and outside traditional treatment settings, working to address social determinants and improve health across socioecological levels [12,13].

Theory that supports peer-based interventions suggests that shared characteristics and experiences between peers and their communities can enhance the effectiveness of traditional public health interventions [14,15]. The assumption is that, as people who are trusted members and/or have a close understanding of the community, peers can better disseminate health information and support people in navigating an increasingly complex health care system [16]. Evidence shows that peers can contribute to behaviour change through different modalities including education, social support, affecting social norms, and promoting self-efficacy and advocacy [17]. Importantly, research suggests that peer-based interventions are *most impactful* for disadvantaged populations who have fewer resources, and experience barriers, including shame and stigma [18,19].

More broadly, peer-based interventions share affinities with integrated care and community development paradigms that focus on providing opportunity for people to become “empowered users of health services and advocates” [20]. Community-based peer programs similarly offer contextual, sensitive and holistic approaches to health and advancing equity. They can also contribute to improve systems through building better understanding among clinicians and service providers of the strengths, needs and general environments of the priority populations they serve [21,22].

While there is growing evidence of the health and wellbeing benefits of peer-based initiatives, the model has been described as a “method in search of theory” [23]. As these programs are implemented in complex settings, how and why activities produce outcomes, in what context and for whom is multilayered. This realist-informed, theory-driven evaluation – grounded in data from a health and wellbeing peer educator program in the Australian social housing context – contributes to developing the evidence base on the underlying factors of success guiding equity-focused integrated care interventions that use peers.

The intervention we report on is innovative on two counts. First, the pilot is an example of Integrated Care program that is ‘people-driven’ [24], meaning *initiated and led* by people with lived experience. The program was community-led from its inception. Developed collaboratively with health services, the program capitalised on community interest in targeted health information and the desire to enhance collective well-being. Second, while peer support and education has mostly been tested in youth settings [25,26] and in disease-specific ways [27,28,29], our pilot had a broader remit. The program was implemented with a diverse adult population in locationally disadvantaged communities, and targeted health and wellbeing holistically. This paper provides detailed empirical insights of the factors that underpin successful co-design, implementation and sustainability of peer-based integrated care program and can inform future policy and program developments.

### Intervention overview

The peer wellbeing educator program (the program) was implemented in the inner-city suburb of Waterloo, in Sydney, Australia. There is substantial socioeconomic disadvantage in Waterloo that is concentrated in the Waterloo public housing estate – a densely populated area located close to Sydney’s CBD and home to about 2,500 people [30]. Waterloo is the largest public housing estate in Australia mixing tall towers and small apartment blocks: it is a very culturally and linguistically diverse place with many residents born overseas, an ageing population, and a significant proportion identifying as Indigenous [31]. The management of government services in the estate is both multilayered and shifting. The local health department, the Sydney Local Health District (SLHD) provides most health services to the area, including community and mental health services, hospital, ambulance, public health and preventative services. There is also long standing and well-established community service sector in the area and organisations committed to support and empower residents.

There are persisting health burden and health inequities in Waterloo, in line with other evidence showing that social housing residents in Australia face health inequities linked to poverty, stigma and chronic conditions [29]. A recent survey of Waterloo residents revealed ongoing issues with physical and mental health and lower life satisfaction compared to averages in the Sydney Region [32]. There has also been concentrated effort to improve integration between services through place-based, equity-focused initiatives and they have shown early positive result [33,34]. However, residents continued to express concerns about gaps in health care, vulnerable transition points, and general barriers when accessing the right services, at the right time and for a cost they can afford, suggesting more targeted action was needed.

The program was developed in response to these concerns, as part of the SLHD’s participation in the International Foundation for Integrated Care (IFIC) Autumn School in 2021. The IFIC Autumn School is an intensive training course that brings together multi-disciplinary working groups to develop structured implementation plans for new integrated care project. A group comprised of housing tenants, SLHD staff, local council and community services representatives, and a health equity researcher participated in the collaborative planning workshop. Through structured needs assessment exercises, the group identified both opportunities and barriers for improving health and wellbeing among social housing residents (see Table 1).

**Table 1:** Co-design phase: identifying opportunities and barriers for better integrated care.


OPPORTUNITIES	BARRIERS

Interest for more relevant and targeted health information	Low health literacy and lack of knowledge regarding health services and pathways

Motivation to support neighbours and community members.	Red tape and navigation issues (patient and service-level factor)

Interest from residents in being more in control of their health and develop capacity to help each other.	Rising levels of social vulnerability and rising levels of complex care needs (e.g. mental health management and addictions)

SLHD committed to place-based working and collaboration with residents.	Increased social isolation post pandemic


Drawing on international integrated care frameworks like the WHO model for integrated people-centred health services [20] and IFIC’s nine pillars of integrated care [35], the group selected a peer educator model as the best way to both leverage community strengths and to support existing services in addressing barriers to health care. Through this process, the program was co-designed to train local people to be information-gatherers and health educators using a peer-to peer model. Housing tenants with lived experience captured the goal of the program under the tagline: “you are not alone and have the right supports” to live a “healthier, more engaged and fulfilled life”. Specifically, the aims of the program were:

1. Improving knowledge about health and wellbeing
2. Empowering residents to manage their own health
3. Reducing social isolation
4. Expanding and illuminating pathways to health services

Implementation began in July 2022. 21 culturally diverse peer educators aged 25–85 years old were recruited and trained. All but two were housing residents in the Waterloo, or nearby social housing estates at the time. The program consisted of two phases. During Phase 1 (October 2022-February 2023), peer educators received core training on the health system and peer skills (e.g. safety, listening and de-escalation), and topic-based training from SLHD experts on health topics that the group had selected, such as mental health, women’s health, oral health, Alcohol and Other Drugs (AOD), or hoarding and squalor disorders. Training was reviewed and amended in the early phase of implementation using feedback from peer educators and staff. Phase 2 (March 2023-October 2024) involved community workshops and education delivered by peer educators. While continuing to adhere to evidence provided in the training, in Phase 2, peer educators were encouraged to be creative in the delivery and the health messages of these workshops. They delivered activities ranging from mental-health-themed trivia nights to more didactic presentations on these topics. They organised hands-on nutrition and diabetes information lunches; a series of affirmative arts-based sessions, a program of work informing residents about decluttering and providing assistance in taking action. Some peer educators also worked alongside health services in outreach efforts for harm minimisation and screening activities (e.g. Hepatitis C testing, overdose reversing drug distribution, and breast cancer screening).

Throughout Phase 2, regular debrief meetings were organised for peer educators to report back to the group and to review the impacts of activities. These meetings were also useful for peer educators to feedback recommendations to improve services (across access, navigation and flow). Peer educators were remunerated in Phase 2 only, when they delivered community activities for a minimum of 3-hour bloc.

## Methods

The Health Equity Research and Development Unit at SLHD – conducted a concurrent, realist-informed evaluation of the program. The study aimed to assess the effectiveness and impacts of the program on community (including peer educators) and health services. The evaluation also investigated how and why the program worked, using a realist-informed methodology. A realist approach focuses on causal explanations, striving to understand the mechanisms that produce outcomes, and how these work within specific contexts [36]. It is particularly useful in complex interventions to help uncover how specific intervention components trigger mechanisms in a given context, leading to the outcomes typically measured in standard evaluations [37]. A realist methodology suited our research objective of generating insights on how the program worked and the full range of impacts it may produce – with the view of informing recommendations for sustainability and transferability.

The study design was based on mixed methods, primarily using qualitative instruments (see Table 2). Semi-structured interviews with peer educators, community-based NGO staff, and health services staff investigated their perspectives on the impacts of the program and the processes or potential drivers generating impacts. We conducted ethnographic observation of training sessions and community activities delivered by peer educators as well as staff debrief sessions. We used document analysis to triangulate and refine our findings, including material from the co-design and implementation phases. In addition to qualitative data, the implementation team routinely collected quantitative process data via survey of peer educators, and recording participation rate and reach in both phases of the program. Other data from community members attending peer-led workshops was not recorded.

**Table 2:** Mixed methods design.


**Mixed-methods study**	**Qualitative data**	**Semi-structured interviews**	14 peer educators5 health and social care staff(July–September 2023)	Analysis – deductive and inductive approach

		**Observations**	Training modules for peer educators (13 × 3 hr) Reflection session with peer educators (2 hr, May 2023) Community-based activities led by peer educators(10 × 1.5 hr) Staff debrief sessions(8 instances; 20–30 min each) Findings validation session with peer educators (1 hr, December 2023)	

		**Documents**	Promotion material Training material Activity reports Correspondence from implementation team members, partner services and organisations	

		**Feedback forms**	Feedback forms completed by peer educators post training sessions (n = 29)	

	**Quantitative data**	**Survey post-training**	n = 11 (2023)	Analysis-Descriptive analysis

		**Participation rate and reach for community-based activities**	Results recorded by implementation team	


Every peer educator who completed Phase 1 was offered the opportunity to be interviewed (n = 19). Staff in health and social care services who had delivered training and/or worked with peer educators were invited to participate (n = 6). We aimed to capture a range of experiences, so we conducted observations of modules delivered by different services, as well as different types of community activities until we reached saturation.

### Data collection

We initially developed hypotheses of how and why the program was meant to achieve its stated objectives using existing literature and discussions with the implementation team. We refined these hypotheses – known as initial program theories in realist approaches – through collaborative workshops with both the implementation team and the larger advisory group for the program. These hypotheses were then used to develop a semi-structured interview guide (see supplementary material 1). The questions were adapted to interview peer educators and staff.

In collecting data, we continued to maintain a clear distinction between the implementation and evaluation teams so that participants would feel comfortable sharing their perspectives, including any criticisms or unintended outcomes. The majority of interviews were conducted face-to-face by the lead researcher (ET) and the remaining by a research fellow also familiar with the program (one interview was conducted online using Microsoft Teams). Interviews lasted between 25 and 92 min, were audio recorded and professionally transcribed. Interviews were conducted in English as the program activities took place in English. Observations were conducted by the same two researchers using prompts from a standard observation guide [38] adapted to the study purpose of identifying mechanisms and impacts against the program’s aims. We observed over 60 hours of program activities, that included a variety of trainers and topics as well community activities of various formats.

We supported the implementation team with developing a survey to measure the effectiveness of the peer educator training (Phase 1, 2023) using scales from validated health literacy and health education questionnaires [39,40]. The survey was administered in person, by the implementation team as part of their Quality Improvement process.

### Data analysis

Interviews were analysed using NVivo 12 Software by the principal researcher (ET) using our initial program theories as a base coding framework. We combined a deductive analytic approach to test and refine initial theories with a more inductive approach attentive to new or unexpected findings. A sample of interview transcripts (n = 5) was cross-analysed with a member of the research team to test validity. Through this process, our coding framework was revised to reflect the ways the data aligned, expanded or challenges our initial hypotheses. In line with a realist approach, we prioritised data that could inform the functioning of the program and how it fitted with the context and diverse people’s strengths and needs. We also looked for alternate explanation and cases, and triangulated information where possible using observation fieldnotes and documents.

### Ethics approval

Ethics approval for the study was granted by the SLHD Human Research Ethics Committee (X22-0328 2022/ETH01687). Written informed consent was obtained for all participants.

## Findings

Over the course of the program (2022–2024), 19 training sessions were delivered and peer educators implemented 29 community workshops and activities in Waterloo and in adjacent areas that present high locational disadvantage with over 650 social housing tenants attending. Project staff indicated participation rates were higher than outreach and health promotion initiatives ran by local district staff. Anecdotally, it was noted that residents who rarely engaged with health or community services (if at all) attended peer-led events, suggesting increased reach.

**Figure 1:**
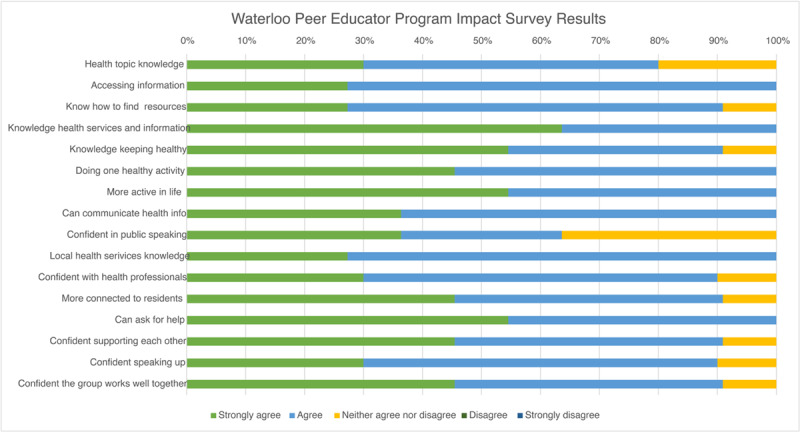
Impacts on peer educators (see supplementary material 3 for survey details).

Of the 19 invited, 14 peer educators participated in an interview (labelled “PE” in the quotes below) and 5 staff members from health and social care services (labelled “S”) (see supplementary material 2 for demographic data). The interviews took place after core training had been completed and each peer educator had delivered at least one activity to their community. Two from the 14 peer educators interviewed had exited the program after training due to personal circumstances (one experienced severe anxiety when delivering public-facing content, while the other had loose community ties due to not living locally or in social housing). and their insights were useful to test the causal mechanisms of why the program worked (or not) and for whom. The survey was completed by peer educators after training (n = 11) to highlight the impacts of the program (Figure 1). Impacts and themes are bolded in the text below.

### Impacts of the program

Data from the survey and interviews identified the main impacts of the program in relation to its objectives of improving health literacy, social connectedness, pathways to services, and general health and wellbeing for residents, as summarised in Table 3. We also investigated impacts on health system that indicated influence of the program.

**Table 3:** Perceived impacts of the Waterloo peer educator program.


**Impacts on peers and community**	Increased active engagement in health and improved knowledge Developed knowledge and personal skills Reduced social isolation, strengthened social support Positive change in attitudes and levels of health behaviours Empowerment and advocacy for health Appropriate trusted and accessible health services (increased uptake of services) Improved health outcomes Positive social outcomes

**Impacts on health system**	Incorporating collaborative and strength-based approaches Tapping into community knowledge and patient experiences to enhance services Building relationships and trust to optimise services’ reach and uptake Activating collaboration and forming cross-sectoral partnerships


At the individual level, the program led to **increased active engagement in health, and building of knowledge and skills for the peer educators**. Over 90% of peer educators said their understanding of how to keep healthy had improved (Figure 1). Knowledge of specific health topics and services also increased, enabling them to do health promotion in the community:

“There was things like domestic violence and family violence… I had lived experience, but I really didn’t know how to get really the supports. So, that was really educational for me… I know how to support somebody… I can actually confidently be of assistance.” (PE1)

“When I have chance conversations with other people, with families and friends…I do recommend, I do tell them, you can go here [to health service].” (PE7)

Benefits included **increased self-efficacy, confidence, and strengthened positive life satisfaction**. All peer educators surveyed reported a more active and positive engagement in life:

“[The program was good] for confidence, for connection, for self-esteem… To add something worthwhile to your day. All those benefit, and all of those contribute to your overall health.” (PE3)

At the interpersonal level, participants reported **reduced social isolation and improved ability to form and engage in networks of support**. As a service provider observed, the program: “creat[ed] situations where people can meet other people and socialise in a meaningful way” (S5). Positive connections with peers at community health events contributed to **improved health literacy** and **capabilities for action**, giving a sense of agency in contrast to the dominant norm in the estate:

“Especially the housing culture, many people give up hope of finding the right [help] ‘oh there are no doctors, and Medicare, and they get into a rut…everything’s bad’. We are able to tell them that you can go to this place to get help.” (PE Reflection session, May 2023)

Peer educators reported they felt more confident to collectively speak up for their rights (81%), as also noted by staff and observed in community forums (observation September 2023), suggesting **empowerment and capability for advocacy** had increased. Other examples included peer educators advocating for community pharmacists to stock overdose reversing drugs, or work with the building management authority to upskill the concierge workforce to support residents:

“it’s to give [the concierge] information about if they’re worried about somebody who they can ring. Presently, all they can do is ring [Department of Community Justice], who know nothing.” (PE5)

Improved confidence and support, combined with health information also contributed to **increased uptake in health and community services** (reported by peer educators and recorded by implementation team) and **positive change in behaviours needed to achieve better health outcomes** (Figure 1). Notably, the program also contributed to **positive social outcomes** with peer educators gaining employment which they attributed to the program’s impact (interviews PE1; PE3).

We also tracked impacts on the health system. Findings indicated signs of positive change in some health services that align with the values and practice of integrated care. Through working with peer educators, services reported a shift towards a more **strength-based and partnership approach with consumers** who belong to vulnerable groups, recognising that “patients and clients have greater skills than you can imagine” despite “the patient-staff relationship [being] still a bit skewed” (S3). Services also noted the value of lived experience and community knowledge in **optimising service delivery in practice**. For instance, a desire for more prevention and early intervention action leading to more screening and health promotion activities. The rebuilding of trust with community post-pandemic was another key impact:

“it does help me with my relationship with them in the community […] since [the training] I’ve actually had them, different peers approach me for different reasons, just for advice on certain things.” (S1)

The program generated **collaboration between services across health and social care** optimising service reach and uptake. The “declutter workshop” and “improving safety and living conditions” initiative illustrates this well. It was designed as community education and wraparound support to tackle hoarding and squalor issues that brought together different health services (mental health, social worker from integrated care, community-based navigator), social care NGO, fire brigade (safety education) and Housing management.

### Mechanisms and factors of impact

**Figure 2:**
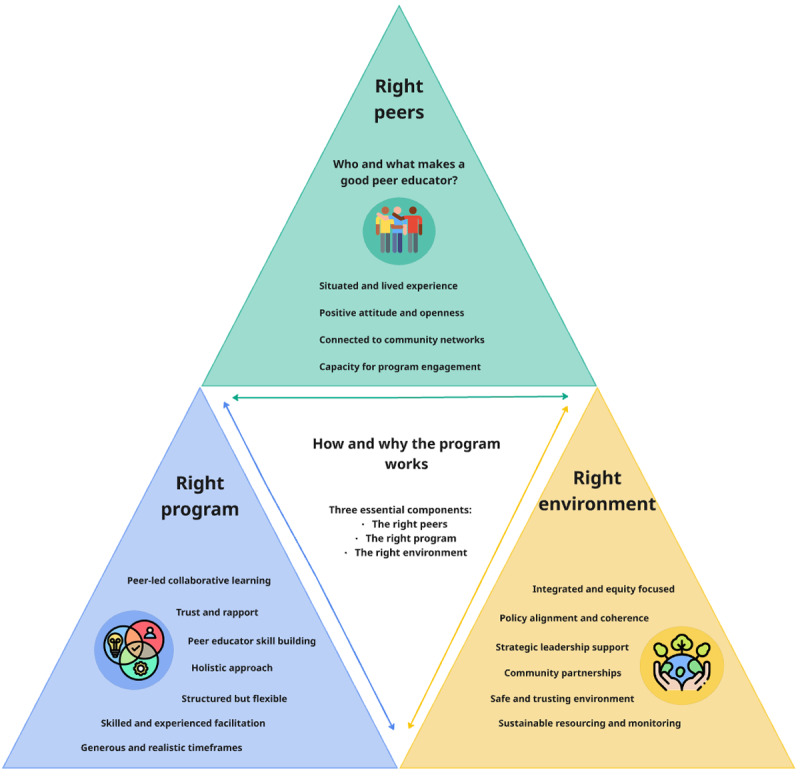
Mechanisms enabling successful peer education program outcomes.

Following a realist approach, we explored the mechanisms, meaning the combination of resources and conditions available in the context of the program, that contribute to producing impacts. We organised these mechanisms across three dimensions: individual characteristics (the right peers), program coordination and training (the right program) and organisational culture and policy (the right environment) (see Figure 2).

#### The right peers: individual characteristics

A core initial assumption was that people are more likely to engage with and listen to an educator who they consider a peer. Peerness, as perceived similarities, in this program is based on participants being social housing residents. We found this **common ground** is a **necessary but not sufficient** condition and there are additional characteristics that make it more likely for participants to become effective peer educators as this service provider emphasised: “It’s not just lived experience, it’s their skills…People think that people who are disadvantaged have no skill and it’s the opposite.” (S3) Specifically, **situated knowledge of the community, lived experience** of particular health, psychosocial, cultural or economic conditions (S3 and S5), and **skills** like the “capacity to absorb insights from others” (S2), were also critical factors and “strengths” for a peer-to-peer approach (S5).

Residents are not passive recipients of the program. What they brought to the table interacted with the context, group dynamic and training, contributing to (or inhibiting) outcomes. We identified **peer qualities** that influence the likelihood of retention, and producing quality peer education and community interaction that related to: a **positive attitude** (change is possible), **motivations** that aligned with the goals of the program, involvement in **social networks** and **capacity to participate in training** (reflexivity and openness).

“We had common ground that brought people together…People want to talk and are interested and are supportive in our group of each other. And I think that flows through everyone.” (PE3)

Importantly, while some aspirations and capacities were apparent at baseline (recruitment), others were more latent and *emerged*. They were realised over time through interaction with the group and through **extensive training**. Peer attributes therefore should not be understood as static – instead the **process of engaging and reflecting** as a group also brought these assets to the fore:

“just being in the group…It’s just a little bit easier to think out loud, I guess, to process things, if you’ve got someone to sound off who’s on the same level of knowledge as you (PE2)”

#### The right program: collaborative learning, equity, time, and support

Initial assumptions emphasised the notion of information for the purpose of health education as an important component of the program reaching its intended goals. Not all information is equal however, and the design of the training program (Phase 1) was instrumental to produce **accessible and acceptable health promotion information**; also setting up the foundation for effective public-facing community workshops led by well-equipped peer educators (Phase 2). The training program adopted a **holistic approach to health and social needs**, with “multifaceted” topics, rather than the more fragmented model of clinical services (PE Reflection session, May 2023). Participants aligned with this integrated approach to health and social care:

“It’s all the aspects of my life…that needs nurturing and that’s been nurtured… we delved on a lot of topics that affect the community. I felt like… I’m bringing up the community.” (PE7)

This holistic approach enabled training that was structured but **flexible**, the message being that of a “**no wrong door**” when it comes to getting health advice or navigation – a message that carried over in the community-facing workshops. The nutrition and diabetes workshops illustrate this well: topics included healthy eating plates but also connections between eating and mental health, and considerations for budgeting, environmental, and social constraints (observation, Feb 2023). Referral to social worker and support for diabetes management were also offered.

“I like the diabetes [workshop] because …diabetes’ not just a physical health problem, it’s also a social, psychological health issue, because diabetes impacts your social relationships, and your experience of the disease is also affected by the people around you.” (PE7)

Awareness of people’s wider social and community context therefore also informed the kind of solutions or pathways to health presented in workshops. Both peer educators and health staff recognised that prescriptive approaches and medical recommendations tend to be ignored in low income and marginalised communities like Waterloo. Instead, peer educators worked with health experts to develop information that offered **diverse course of actions adapted to people’s circumstances and their self-defined needs and priorities**. As a corollary, the peer-led approach meant the program **tackled the inhibiting effects of both health and poverty-related stigma** “around people living in housing” (S5). Peer educators built on their own **personal stories, emphasising “small changes”** (PE2) in the information delivered:

“If you’ve got experts talking to you, it’s like, ‘Oh, housos [colloquial Australian term for social housing resident] don’t know anything, we’ve got to tell you.’ Whereas, if I’m a houso talking to housos, it’s like… we are real people looking after ourselves…you can hear from people and tell people how *you* have changed, so you know that change is possible. It’s not saying Hey, you should be doing this.” (PE2)

“We can pass on the information we’ve learnt, and be a liveable, visible connection, that’s where the power is; living, seeing, believing… it doesn’t have to be a huge thing; it could be a little thing to make it good for everyone.” (PE3)

At the same time, **information in and of itself is not enough**. People need to be able to appraise the information, relate it to their own situations and feel capable to take action. In other words, **relational learning and confidence-building** need to occur as well. We found that collaborative learning activated through **participation and equity** were key factors of success identified by both service providers and peer educators. Comments about “acceptance”, “mutual respect” (PE4), “humility”, and “two-way” learning (PE11) also illustrate how traditional power differences were disrupted.

“It was about trying to upskill people…you’ve got to be careful with all of it that it’s not patronising.” (S5)

“We could relate [to the information] But what’s important is that it shouldn’t be all one way. So it should be two ways.” (PE11)

“It’s been a bottom and a community-led thing about what’s the most important areas for community and what we really want to get involved in as well. That’s the key to why it’s so successful.” (PE10)

“That’s the thing about peer education, you’re all equal and if you want, you can go to the expert, but you don’t have to unless there’s something you don’t know.” (PE2)

This process can look “messier” (in the words of a service provider), but it creates a **safe and supportive environment** (PE reflection session, May 2023) as well as space for trial and error which becomes part of the social learning process: “I can say something and didn’t matter if it was right or wrong, I was there to be able to share, and my voice mattered… that helped me” (PE1). This relational aspect of the program that co-produces information for better health, and fosters capabilities is underpinned by **trust** and this is also a **prominent mechanism for successful engagement and program sustainability**. Brokering trust in a context of high social vulnerability and high staff and service turn over can be challenging however: “Rapport and trust take a long time to build” (S1). We found that **engagement that is proactive, sustained and unhurried** was crucial to create and maintain that trust.

This type of engagement requires **time and resources** particularly in the early stages of the program with **generous and realistic timeframes**: “not expecting people to be work ready and deliver” immediately but giving time to build confidence and a sense of a collective ownership (S3; observations 2023). Residents were more likely to trust and invest in the program because of this consistency, with regular and easily accessible sessions and staff:

“[Health and social care staff] they walk out, once they get promoted…they never come back…Off they go. So basically that’s why the peer education works, because we’re local. [It’s like] I sign a contract saying I will not leave. I mean, you have to build up that relationship [with other residents].” (PE12)

**Informed discussions and feedback** also helped to clarify **expectations** and was an important part of the process to maintain trust (observation staff debrief July 2023; S3 interview). There is a risk that community activities can become a “forum to air complaints” and reinforce “hopelessness” (observation, staff debrief Dec 2022). In contrast, being **genuine and transparent** about what the program can achieve while reinforcing the idea that residents have agency to effect *some* change was essential, with participants describing **program facilitation** that “so friendly, and so supportive and so optimistic” (PE2).

Lastly, **additional material and emotional support** was also needed to overcome barriers to engagement in both training and in community-facing workshops. An experienced program lead from the local health district (who is also the area’s community-based navigator) and the manager of the local community service organisation worked to provide ongoing affirmational, instrumental and logistical support throughout implementation.

#### The right environment: Organisational factors and culture

Although organisational factors work in the background and are not always considered in explicit and tangible ways, they are crucial to programs’ success and sustainability.

**Integrated and equity-focused portfolios** like “Priority Populations and Places” and “Integration and Partnerships” – where the peer educator program is managed – that sit outside of a single facility or clinical stream are important enablers of activity occurring across multiple areas within the health systems, in response to community needs. We also found that **leadership and senior buy-in** in these portfolios increased the credibility of the program and encouraged participation from other staff members and services. **Supporting policy and strategic plans** that centre participation, empowerment and patient-centred care also provided critical levers for the inception, implementation and resourcing of the program.

Beliefs, values, and priorities in an organisation also impact the implementation of programs. We found that the implementation **team’s culture – equity, strengths and place-based –** was key in enabling a community-led approach to health promotion and to deliver culturally appropriate information to support behaviour change. This way of working focusing on parity, collaboration, and community development is not shared across all parts of the health system but there was a **deliberate and systematic** effort to impart it to other stakeholders interacting with the peer educator program (for instance through guideline documents and briefing from the program lead).

“The trainers [from health services] need to be trained.” (S1)

“There is stigma around people living in housing…but you’ve got all these people that are obviously intelligent and resourceful” (S5)

**Strong and early partnership with community-based organisations** like community service providers, charities and food aid, that share these values was also important to embed and amplify positive program outcomes within and beyond health system pathways. Lastly, **quality monitoring and evaluation** using metrics that are relevant and suitable to the program was also key to demonstrate success.

## Discussion

Our case study in an inner-city suburb with high levels of deprivation describes how a community-driven health and wellbeing peer educator program can support social connections, illuminate appropriate pathways to better health (including via health services) and improve health and wellbeing. Our realist-informed evaluation based on interviews with peer educators and service providers, extensive ethnographic observations, and a short survey showed that a combination of the right peers, the right program and a conducive environment can support the delivery of an equity-focused integrated care program to people living in public housing with great health and social need. There is increasing interest in community engagement and peer-based programs as a way of tackling entrenched health inequalities. Our study contributes empirically rich and theory-supported insights to this field by illustrating the key causal mechanisms underpinning community-based peer education that supports health and capabilities. An important lesson learned from this program is to maintain a community-led approach from the design phase through to implementation, knowing that the process may not always be linear or predictable and requires flexibility and time for genuine thinking, experimenting, and collective decision-making. At the same time, it was essential for the program to be located within a supportive institutional environment with resident peer educators equipped with the necessary resources and guidance to deliver action. Through this community-led approach, the program also encouraged health promotion strategies that extended beyond standard information provision, emphasising a targeted and tailored approach for optimal outcomes.

This study extends realist evaluation theory in community health contexts by demonstrating how realist methodologies can systematically capture multi-level impacts across individual, program, and system domains – moving beyond traditional peer program evaluations that focus primarily on participant outcomes. There is growing recognition that evaluating the structural effects of these programs on health/social care systems is important and there is burgeoning research in this area that uses systems thinking to map and measure these effects, including through developing indicators [14]. Contributing to this emerging field, our evaluation purposefully examined not only impacts on individual community recipients, but also the program’s role in influencing, informing and building system capacity to adapt and respond better, knowing that *embedding* equity positive impacts requires change and resources within systems.

Our findings align with published literature in terms of substantiating the important role of peerness that brings with it situated knowledge, cultural competence, and trust [18,27]. However, we also found that peerness is necessary but not sufficient and identified properties such as motivation, attitude (sharing commitment to help others and understanding of social and structural determinants), and substantial training as key mechanisms of success. Put differently, lived experience and shared characteristics is important to help design and deliver an appropriate and accessible intervention but additional screening, training and mentoring of the peer cohort is needed to maximise and sustain equity positive outcomes.

Our study also found that a flexible, holistic approach that is responsive to the complexity and specificity of people’s social world, material conditions, and life circumstances is key to deliver effective health promotion information and finding ways to make behaviour change and services available to people living in low-income communities. This is in line with literature on strengths or asset-based approach to care that centres accessibility and ensuring supportive milieux so that people can focus on what improves their health and wellbeing [41]. Another significant element of the program was to have a facilitator knowledgeable and embedded in the local area working in tandem with a community-based organisation so as to maintain a focus on community strengths and needs. Existing research into interventions aimed at supporting action for equity similarly shows that solutions are *not* to be found in acontextual lifestyle interventions, but rather in place-based, locally-driven initiatives [42].

At the same time, this focus on the local in our pilot reflects a particular sharing of power between communities and professionals from the health and social care sector. The peer educator program uses a community engagement approach that is critically oriented towards community empowerment as a way of improving health and wellbeing and reducing health inequities. By developing individual and community capabilities in Waterloo, the program promoted different dimensions of empowerment [cf. 42] namely: “power within” through individual self-esteem, confidence, and efficacy.; “power with” by building social connectivity; and “power over” by addressing resources and building advocacy. Legitimising community input however also requires a *giving over of power* to people in social housing communities so they can enact changes across the health and social care systems that serve them. In the context of health systems that value professional credentials and medical expertise, this is unusual and can be challenging, as also observed in the literature on peer workers and navigators [6,15].

This study also demonstrates the power of realist methodologies to capture system change and its process. We found that system receptiveness (i.e. intangible elements like a culture oriented to social care and equity) as well as provision of opportunities and resources were crucial factors. While our study showed some indication of system-level impacts – through health staff valuing lived experience perspective and strengths for instance or forming new collaborations – developing further empirical measures of impacts on systems will be an important component of the program’s next iteration.

Relatedly, our study prioritised qualitative methodologies and a theory-driven, realist-informed approach to evaluation. This was deliberate, and we argue, a key strength of our research. Moving beyond questions of effectiveness, this methodology was instrumental to delve into the complexity of the program to explore in what ways, and for whom peer education works in the context of social housing communities, and for capturing a broader range of effects, including on systems. Running the evaluation concurrently to implementation with extensive data collection and a research team embedded in the health district allowed for rich observational data, as well as supporting the program to make changes in the early phase of implementation. Not using predefined metrics of success and rather working closely with the implementation team to examine what success would look like, fostered greater buy-in for the evaluation component of the program. Overall, this approach also allowed us to generate insights and recommendations that are specific enough to be useful for transferability (see Figure 3).

These findings have implications for future initiatives and research. Developing additional quantitative measures of impacts on health outcomes could be useful to support health services needing to demonstrate quantifiable outcomes. Future research could also broaden the scope of investigation to understand the range and mechanisms of impacts for community members attending peer-led sessions; alongside exploring system-level impacts as already mentioned. Building more opportunities within health and social care systems to support and implement this type of peer and community-led program is important if we are to see sustained change.

## Lessons learned

**Figure 3:**
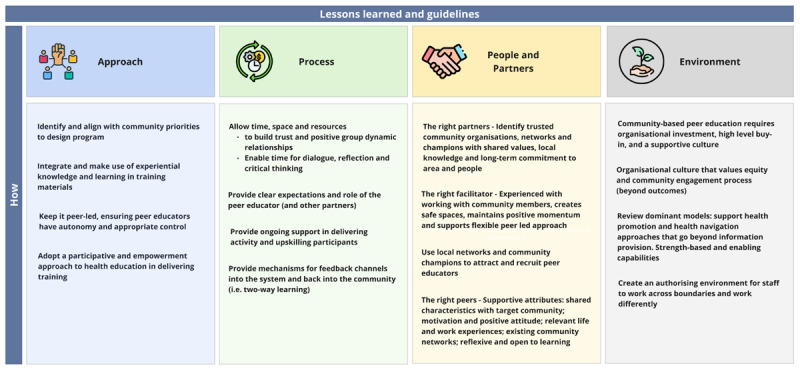
Lessons learned (practice focused).

## Conclusion

Peer and community-based initiatives are becoming an increasingly important component of integrated care programs, acting as a bridge between community members and services so they can deliver more equitable and appropriate care. Our evaluation of a pilot program of peer educators working in a locationally disadvantaged area shows that important progress can be made in terms of health, wellbeing, social connection and accessible services. When motivated, adequately trained and supported community members lead the design of solutions for issues they have identified, meaningful empowerment can be achieved with flow-on effects for health and wellbeing. Our research illuminates the multi-level success factors and mechanisms enabling peer education initiatives to succeed providing insights for similar initiatives in diverse community contexts. People-driven peer education programs can advance both health equity and integrated care by placing communities at the centre of both design and delivery, creating sustainable pathways for change that reflect local contexts and priorities.

## Additional Files

The additional files for this article can be found as follows:

10.5334/ijic.9102.s1 Supplementary Material 1. Initial Program Theories and Interview Guide for the peer educator program.

10.5334/ijic.9102.s2 Supplementary Material 2. Interviewees characteristics.

10.5334/ijic.9102.s3 Supplementary Material 3. Impact of the Waterloo Peer Education Program.
