# Supplementary material for: By Residents, for Residents: Evaluating a Community-Led Peer Health Education Program in Australian Social Housing Communities
Source: Int J Integr Care. 2025 Nov 5;25(4):5. doi: 10.5334/ijic.9102 (PMC12594079; doi:10.5334/ijic.9102)
Supplement: Supplementary Material 1. — Initial Program Theories and Interview Guide for the peer educator program. [file ijic-25-4-9102-s1.pdf]

Supplementary material 1: Initial Program Theories and Interview Guide for the peer educator program

**Initial program theories (IPT)**	**Context/Hypothesis**
**IPT1. Education to improve health literacy through tailored message and trusted connections with community**	*If* peer educators share *similar characteristics* as the target population and have *experiential knowledge*, they can *act as trusted intermediaries* between health services (HS) and residents, delivering health messages to community participants and increasing health literacy. Better health information and understanding – including understanding of health services and pathways – is expected to contribute to improved health and wellbeing and therefore reducing inequities.<br><br>*We assumed* that peer educators based in the community are more likely to be trusted by other community members and can *deliver more targeted, appropriate and acceptable messages* about health and services available, with the support of HS stakeholders. As members of the community with lived experience, they can help to *demystify and de-stigmatise certain conditions or behaviours.*<br><br>Developing interactive and critical literacy can support a reduction of health inequities in the Waterloo estate by supporting people in taking action for their health and wellbeing.
**IPT 2. Reducing social isolation and building supportive networks to promote better health literacy and outcomes, and reduce health inequities**	*Context:* People in social housing who are socially isolated because of their age, ability, health issues, sexuality, language barriers, or other factors can face difficulties in managing the demands of everyday life. Increased cost of living pressures and curtailed opportunities for social interaction and community activities/services in the wake of the pandemic have compounded

	economic and social difficulties for many. Social isolation can deter people from seeking the help they may need , meaning it has negative implications for health-seeking and health-directed behaviour. It can also make appraising and acting on health information more challenging when there is no relay to do this collectively.<br><br>*If* the program provides opportunities for *building relationships* between social housing residents through structured training workshops (stage 1) and events with the larger community (stage 2), *involving sharing stories, tips and meaningful information*, it can contribute to reducing social isolation and exclusion, build or expand social/support networks, with positive consequences for health literacy and health outcomes.
**IPT 3. Empowering residents to self-manage their health and highlight gaps**	*If* the program maintains *community ownership* (i.e. control and autonomy) over needs prioritised and course of action; and *recognises and values peer educators' knowledge and skills*, participants will be able to use resources from the health system to improve their understanding and confidence and be more in control of their health. Over time and as a group, they may feel more empowered in advocating for change.<br><br>The connection with influencing service practice and highlighting gaps was identified but not fully fleshed out in developing this IPT.

INTERVIEW GUIDE – PEER EDUCATORS	
**Recruitment and reasons for participation**	
1. Thinking back to when you first heard about the peer educator program, what made you **decide to take part?**	*How were you recruited?*<br><br>*What were you hoping to get out of it?*
2. At the time, did you feel like you **had enough information / a good understanding** of health and wellbeing?	*What were your views about health and wellbeing?*
3. At the time, what were you most concerned about in relation to the Waterloo community and the health and wellbeing of people in the community?	
**Overall experience**	
1. Can you tell me first what your experience with the peer educator program was like **overall?**	
2. What aspect did you find more **useful** and **beneficial** in relation to your own motivation and goals?<br>⇨ Why?	
**Training**	
1. How did you feel/ what did you think about the training your received?	
2. To what extent did it **prepare you** for your work as peer educator?<br>⇨ How?<br>⇨ Why/why not?	*Have you used the skills and techniques you learned?*<br><br>*What was your experience of that?*
**Health education activities**	
1. You have done several activities with the community …What were the **challenges of doing that work**?	
2. What kind of **support** did you receive before, during and after delivering health education activities?	
**Impacts**	
1. Do you think anything has changed **mentally** or	○ *Wellbeing & quality of life*

**physically** for you because of taking part in the program?	○ *active engagement in life*<br>○ *attitude towards healthy behaviours + levels of health-directed behaviours*<br>○ *confidence in oneself + confidence in seeking support*<br>○ *sense of agency and control*<br>○ *self-monitoring and insight to manage your health condition*<br>○ *accessing + navigating health services*<br>○ *goal attainment*
2. What has been the impact of participation in the program on your **emotional wellbeing**?	*Any topics particularly helpful?*
3. What has been the impact of **connecting with other members** of the community (peer educators and broader)?	○ *Any friendships formed?*<br>○ *Information shared?*<br>○ *Learn from each other?*<br>○ *Any other examples?*
4. What are your views about health and wellbeing now?	
5. To what extent has your **knowledge of health information** / knowledge of **health services changed**?<br>⇨ Have you recommended any of the services to others?	
**What worked** We have some ideas about what makes the peer education program work for in promoting health and wellbeing, social participation and contribute to improve health services. Of course, the program works for many people but not for everyone.<br><br>Can I tell you those ideas and you can tell me your views on them	
**a.** Peer educators are more likely to have an impact on community health behaviour and beliefs because they have **shared experience/characteristics and build trust with people**	*Was that your experience or were there other factors (e.g. offering other kinds of support?)*
b. If there is **stigma, shame and negative perception** of certain health conditions or behaviours in the community, people are less likely to engage with the program and peer educators need to understand and overcome these barriers.	*Was that your experience, how did you navigate these issues?*

c. **Actively involving peers** as part of health education makes **messages more relevant** to the needs, context, and norms of the community.	*What do you think about that?*
d. **Social connections** are an incentive for some people and may add to the program's benefit, even giving a sense of group belonging.	*Was that your experience?*
**e.** The peer educator program works best when it gives people **confidence and skills to advocate** for themselves and their community, and **health services are willing to listen and respond.**	*Are both aspects important? Equally important ?*
**Thinking about the future**	
1. How do you think program could **be improved in the future?**	*Was there anything you found that the program couldn't assist with or could have done better?*
2. Do you see yourself **carrying on being a peer educator** and in what capacity [*delivering health sessions, or less formal activities*?]	
3. Anything else you would like to **add t**hat might help us to improve programs and to support residents manage their health and wellbeing?<br><br>Any other comments /things I should have asked you	*Future groups/activities?*<br>*Feedback*
