# Supplementary material for: By Residents, for Residents: Evaluating a Community-Led Peer Health Education Program in Australian Social Housing Communities
Source: Int J Integr Care. 2025 Nov 5;25(4):5. doi: 10.5334/ijic.9102 (PMC12594079; doi:10.5334/ijic.9102)
Supplement: Supplementary Material 2. — Interviewees characteristics. [file ijic-25-4-9102-s2.pdf]

Supplementary material 2: Interviewees characteristics

Characteristics of peer educators interviewed

**Code**	**Age and gender**	**Length of residency in social housing**	**Ethnicity**
PE1	45 M	>2.5 years<br>lives alone	South East Asian Australian
PE2	61 F	>30 years<br>Lives alone	White Australian
PE3	63 F	>30 years<br>Lives alone	White Australian
PE4	69 F	>8 years<br>Lives alone	White European Australian
PE5	84 F	>40 years<br>Lives alone	White European Australian
PE6	80 M	>6 years<br>Lives alone	White Australian
PE7	63 M	>3years<br>Lives alone	South East Asian Australian
PE8	67 F	Does not currently live in social housing	First Nation Australian
PE9	70 M	>20 years<br>Lives alone<br><br>Exited the program	White Australian
PE10	57 M	>5years<br>Lives alone	White Australian
PE11	30 M	Does not currently live in social housing<br><br>Exited the program	South Asian
PE12	60-70 F	>6years<br>Lives alone	East Asian Australian
PE13	45 F	>3years<br>Lives alone	First Nation Australian
PE14	60-70 F	Not provided<br>Lives alone	White Australian

Characteristics of stakeholders interviewed

Code	Age and Gender	Organisation and time in role
S1	40-50 M	Health services >10years
S2	30-40 M	Community services > 4years
S3	50-60 M	Health services >5years
S4	40-50 F	Community services >5years
S5	30-40 F	Health services >4years
