# Supplementary material for: By Residents, for Residents: Evaluating a Community-Led Peer Health Education Program in Australian Social Housing Communities
Source: Int J Integr Care. 2025 Nov 5;25(4):5. doi: 10.5334/ijic.9102 (PMC12594079; doi:10.5334/ijic.9102)
Supplement: Supplementary Material 3. — Impact of the Waterloo Peer Education Program. [file ijic-25-4-9102-s3.pdf]

Impact of the Waterloo Peer Education Program

June 2022 – February 2023

To help us understand how the Waterloo Peer Education Program impacted you, please complete this survey and return it to Shane Brown. Your responses are anonymous and this information will be used to help us improve future iterations of the program.

After completing the Peer Education Training Program, how much do you agree or disagree with the following statements?

	Strongly agree	Agree	Neither agree nor disagree	Disagree	Strongly disagree
I have good knowledge about the health topics we covered					
I have enough information to help me start a conversation about the following health topics:					
Alcohol and other drugs					
Addiction					
Mental health					
Oral health					
Nutrition					
The Australian Healthcare System					
Domestic violence					
Finding a health service					
Diabetes					
Population health					
Blood borne viruses (e.g. Hep C, HIV)					
I know how to get information about the health topics we covered in the workshops					
I can get the resources I need to deliver a workshop to the community					
My knowledge of health information / health services has improved					
My knowledge on how to keep healthy has improved					
I do at least one activity, or follow one tip that we discussed in the workshops, to improve my health					
My participation in this program has meant that I am more actively involved in life					
I have gained effective skills to communicate health information to others					
I am more confident about speaking in a large group of people					
I have better knowledge about the health services available to me and fellow residents, and how to access them					
I feel more confident communicating with my doctor and/or other health professionals					
I feel more connected to other residents					
If I need help or advice, I can ask for it					
I feel confident that Peer Educators can work together to support each other					
I feel confident that Peer Educators and their neighbours can work together to speak up for their rights					
The Peer Educators work well together					

Please turn over

Please write a response to the following questions:

What do you feel has changed mentally or physically for you as a result of taking part in the Peer Education Program?
What has been the impact of participating on your health and wellbeing?
What has been the impact of connecting with other members of the community?
How has your knowledge of health information / health services changed?
Have you recommended any health services to others?

Thank you!
